# Fast and Comfortable Robot-to-Human Handover for Mobile Cooperation Robot System

**DOI:** 10.34133/cbsystems.0120

**Published:** 2024-08-13

**Authors:** Chongxi Meng, Tianwei Zhang, Da Zhao, Tin Lun Lam

**Affiliations:** ^1^ School of Science and Engineering, The Chinese University of Hong Kong, Shenzhen, China.; ^2^ Shenzhen Institute of Artificial Intelligence and Robotics for Society, Shenzhen, China.

## Abstract

In this work, we present a method that enables a mobile robot to hand over objects to humans efficiently and safely by combining mobile navigation with visual perception. Our robotic system can map its environment in real time and locate objects to pick up. It uses advanced algorithms to grasp objects in a way that suits human preference and employs path planning and obstacle avoidance to navigate back to the human user. The robot adjusts its movements during handover by analyzing the human’s posture and movements through visual sensors, ensuring a smooth and collision-free handover. Tests of our system show that it can successfully hand over various objects to humans and adapt to changes in the human’s hand position, highlighting improvements in safety and versatility for robotic handovers.

## Introduction

Robot interaction has become a cornerstone of contemporary society, with its applications permeating diverse sectors including manufacturing, healthcare, and personal assistance. In recent years, the research community has increasingly concentrated on tasks that involve collaborative manipulation between humans and robots, as evidenced by a growing body of literature [1–3]. A critical component of this collaboration is the seamless transfer of objects from robots to humans, a fundamental function that underpins successful joint task execution. These tasks frequently demand that robots adeptly navigate multifaceted environments to hand over objects to their human counterparts—a scenario that is steadily becoming commonplace.

Imagine a scenario in which a human operator is engaged in the intricate task of assembling furniture and requires a specific tool. In this context, an assistive robot should have the capability to retrieve and hand over the necessary tool to the operator. Another instance might involve a mechanic who, while working beneath a vehicle and constrained in movement, requests a tool. In such situations, where the mechanic’s mobility is importantly restricted, the robot must hand over the tool with exceptional care. These interactions, where objects are passed between robots and humans, are commonly referred to as robot–human object handovers, a critical function in the broader scope of robot–human interaction. Nevertheless, achieving a handover process that parallels the efficiency and fluidity of exchanges among humans remains a formidable challenge for the robotics community.

The impetus for a robot-to-human handover is derived from the human’s need to acquire an object for a specific task. The object in question may be situated within the robot’s immediate operational area, such as an operating table, or it may be located some distance away. Consequently, the robot must execute a series of actions: navigate effectively and safely to the object’s location, secure the object, and then return to deliver it to the human collaborator.

The handover navigation process is bifurcated into two sequential stages: the initial detection and acquisition of the object, followed by the robot’s traversal to the human recipient. This sequence encompasses four core components: localization, exploration, object grasping, and path planning. A multitude of algorithms cater to indoor localization, leveraging various sensory inputs such as lasers, inertial measurement units (IMUs), and cameras. Of these, laser-based methods [4–6] often achieve superior precision over their vision-based counterparts, primarily due to their inherent ability to measure depth directly and their resilience to fluctuations in lighting conditions [7].

Advancements in exploration algorithms have seen a significant rise in recent years, with high success rates in structured environments. These improvements are largely attributed to sophisticated learning or optimization techniques [8,9]. The field of mobile robot motion planning within structured settings has been thoroughly investigated, with a wealth of research contributing to the field [10]. A common strategy in mobile motion planning involves a dual-layered approach: A global planner [11] first charts a coarse trajectory from the starting position to the intended destination. This is then refined by a local planner [12–14], which processes the global path into a fine-grained, executable sequence, thereby ensuring that the robot’s movements are both precise and responsive. Single-object grasping has been comprehensively studied and now represents a well-established domain within robotics research, as detailed in works such as [15–17].

In robot–human interaction, the physical handover phase is critical and encompasses several key tasks: identifying the handover location, executing the transfer movement, recognizing the human recipient, estimating their pose, tracking the movement of their hand, and ensuring that the robot’s arm moves in a collision-free and safe manner. For human recognition and keypoint extraction—essential for effective hand tracking—both YOLO and MediaPipe [18,19] provide robust and rapid solutions. Furthermore, certain approaches [20,21] utilize two-dimensional (2D) semantic segmentation to delineate the human hand, body, and surrounding environment, mitigating the risk of the robotic arm colliding with the person during the handover. However, these methods are often computationally intensive and, when translating the segmentation results into 3D space, the absence of depth values for points outside the camera’s field of view poses a challenge for collision-free motion planning. Model-based human body and hand reconstruction techniques [22,23] offer a solution to this problem.

**Fig. 1.**
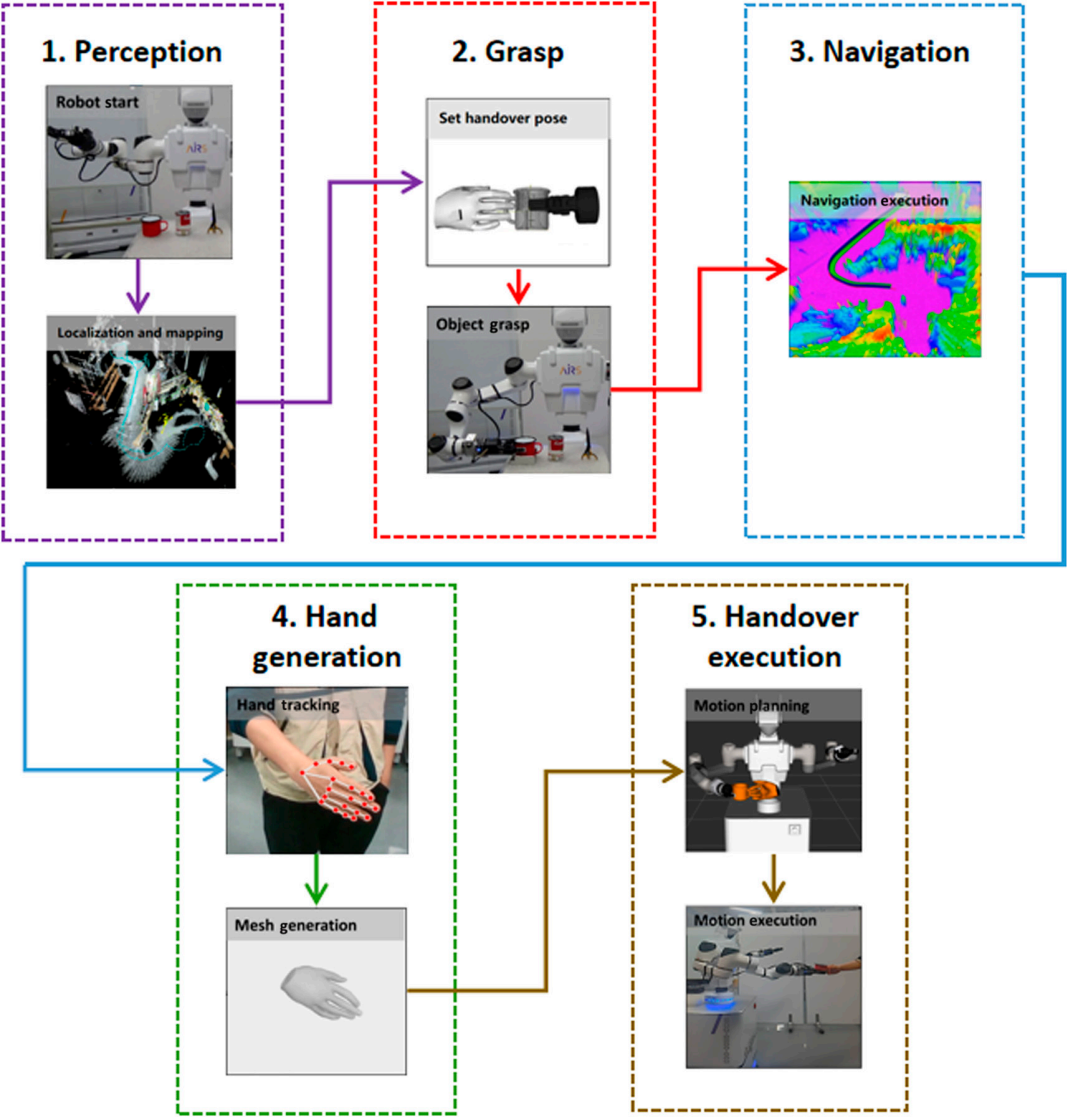
The proposed mobile robot handover system is delineated into five distinct modules: Perception, Grasp Planning, Navigation, Hand Pose Generation, and Handover Execution. Initially, the Perception module enables the robot to search and identify the object within its environment. Leveraging prior knowledge, the Grasp Planning module predicts an appropriate hand pose for successful object grasping. Following this, the Navigation module guides the robot toward the human recipient, dynamically adjusting to their current posture. The Hand Pose Generation module is responsible for tracking the human hand and generating a detailed mesh model, which aids in pinpointing the optimal handover location. Finally, the robot completes the handover by transferring the object to the human using a responsive strategy that adapts to real-time human movements.

As depicted in Fig. 1, our robot, when initiated in proximity to a human, activates its localization module to determine its own pose and incrementally map the environment in search of the target object. Upon locating and securing the object, the robot navigates back to the human to facilitate the handover. It then computes the human hand’s real-time pose to determine the optimal handover point for an untroubled transfer. Throughout the robot arm’s trajectory, we ensure collision avoidance by employing reconstructed models of the human body and hand.

## Materials and Methods

### Localization and mapping

**Fig. 2.**
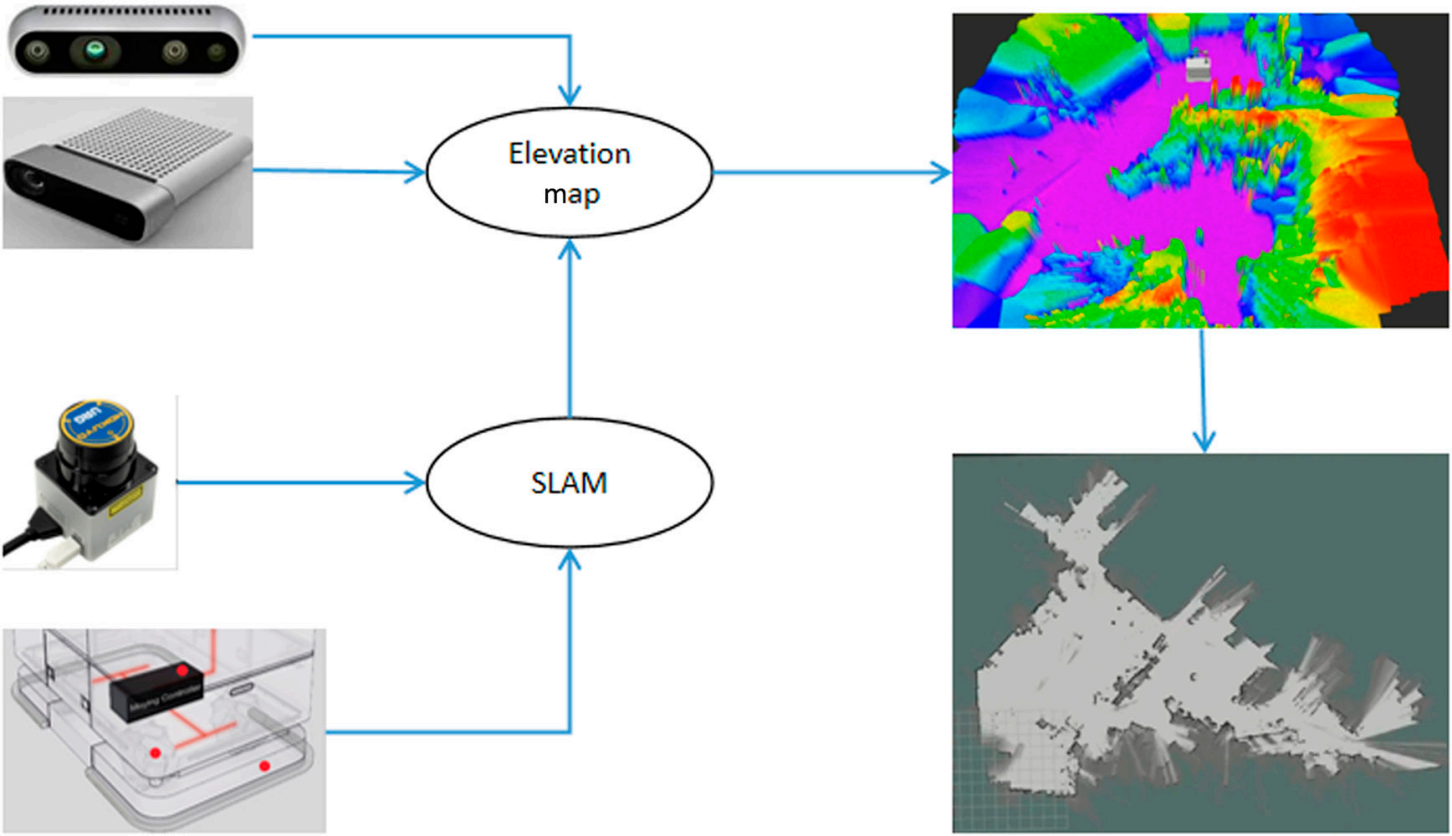
Flowchart of localization and local mapping: Fusion of 2D laser data with the wheeled odometry for SLAM implementation, complemented by depth information from an RGB-D camera to construct an elevation map. This is integrated with the laser point cloud to produce the comprehensive grid map employed for robot navigation.

During autonomous navigation and object retrieval missions, robots must perform real-time self-localization to ascertain the object’s pose relative to the map frame with precision. Our perception system pipeline, which facilitates this process, is depicted in Fig. 2. Our research harnesses lidar-inertial odometry (LIO) technology to underpin the robot’s real-time localization capabilities. This technology undergirds robust global pose estimation, culminating in the synthesis of consistent global point clouds.

In terms of environmental mapping, we embrace the grid map as our preferred representation, which conceptualizes the environment in a 2D array with each cell reflecting a distinct elevation. This 2.5D mapping strategy yields two salient benefits: First, it substantially expedites the search process within a 2D paradigm by curtailing the degrees of freedom, thereby diminishing access times compared to volumetric 3D point clouds. Second, incorporating elevation data into the grid map distinctly outlines navigable zones, a detail of utmost importance for the path planning of mobile robots.

The robot’s capacity to traverse particular areas is gauged by a predefined height threshold within the grid map. Cells that exhibit a height differential within this threshold are designated as impassable. This approach not only simplifies the computational burden associated with environmental navigation but also bolsters the robot’s proficiency in safely and effectively maneuvering over diverse terrain.

### Exploration planner and path follower

Our exploration planner is designed with the primary objective of locating a target, mirroring conventional scene coverage tasks in certain aspects. The robot employs the YOLO [18] object detection system in real time to identify objects it aims to grasp. Once an object is identified, it signifies the completion of the explorer’s task. Drawing inspiration from the methodologies detailed in the Frontier-based approach [24], our task execution is bifurcated into two distinct stages. The first stage is aptly termed Exploration. Most existing algorithms [25,26] encompass this stage, predominantly leveraging rapidly-exploring random tree (RRT) or rapidly-exploring random graph (RRG) to extend local observation points. Subsequently, the Relocation phase is initiated. In this stage, the robot, surrounded by previously mapped regions, needs to be redirected to distant unexplored territories for continued exploration. Throughout this phase, the robot traverses solely within known regions, eschewing the collection of any unfamiliar data.

**Fig. 3.**
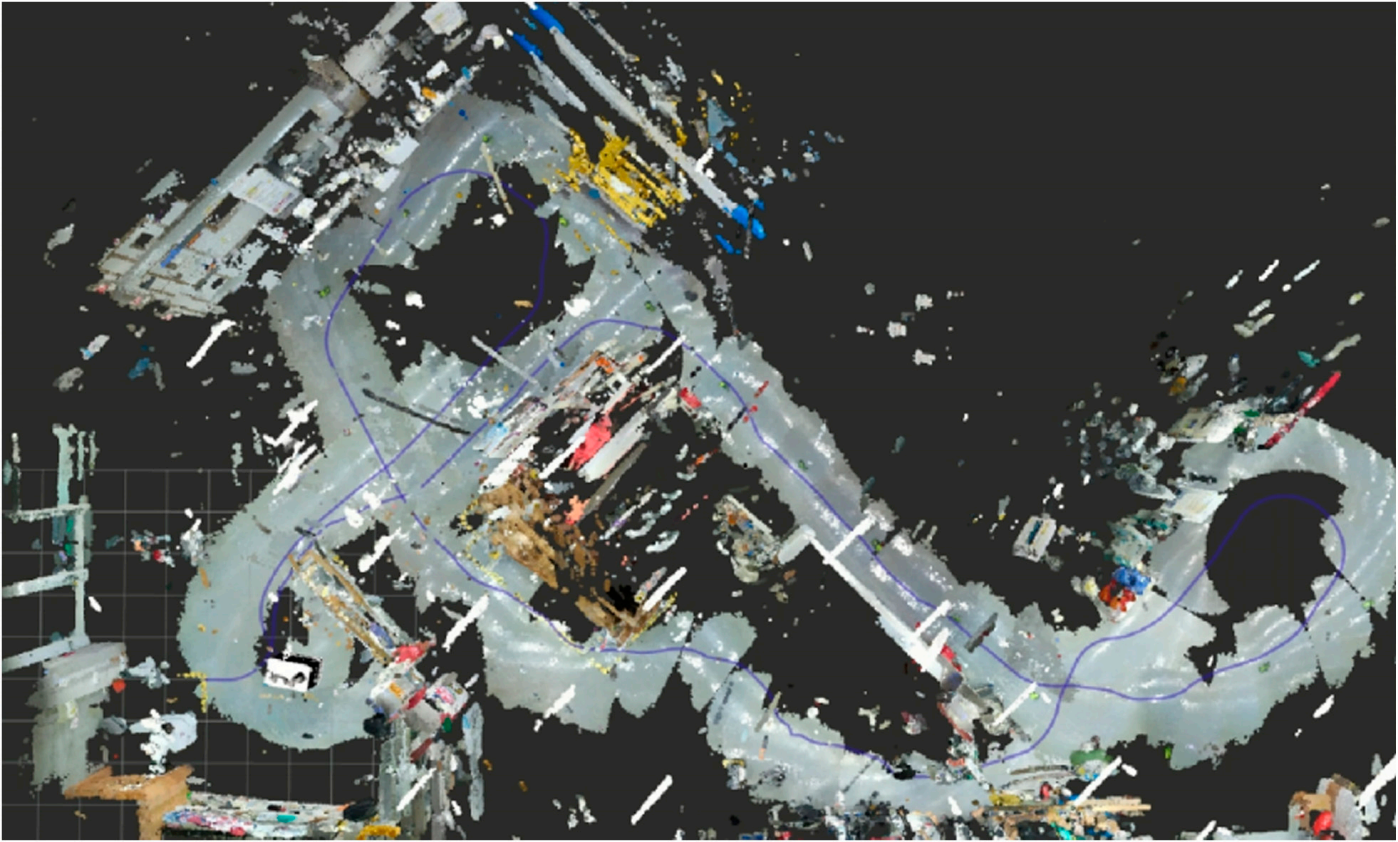
The exploration route map of the robot. The purple line represents the robot’s movement trajectory during exploration. It is evident that the robot navigates along the edges of areas with large obstacles and avoids open spaces, achieving full coverage of the entire environment.

In order to optimize object detection efficiency during the Exploration phase, the robot exhibits a proclivity for scattering observation points in proximity to large obstacles shown in Fig. 3. This is predicated on the understanding that objects the robot seeks to grasp, such as utensils or tools, are customarily located atop more substantial structures like tables or cabinets. Consequently, isolated and diminutive obstacles are often overlooked during the RRT exploration stage, enhancing the search efficiency. This behavior aligns seamlessly with human instincts when searching for objects, offering a more intuitive and effective exploration strategy.

Upon identifying the object, the robot determines its motion direction for the local planner based on the object’s bounding box location. This ensures that the robot progresses in the anticipated direction. Subsequently, it examines the depth continuity within the box, and when the depth is measurable, it precisely estimates the object’s 3D pose to serve as a waypoint for the local planner. Incorporating ideas from this research [9], we have integrated obstacle avoidance with the local planner. By processing point cloud data, obstacles in the robot’s path are identified. Following this identification, paths that are obstructed by these obstacles are eliminated. Among the remaining traversable routes, a selection process pinpoints the most suitable local path to be taken.

### Handover pose and object grasp

To address the grasping challenge, our solution comprises three stages. Initially, tools like Graspnet [16] are employed to generate feasible grasping poses on the object. Subsequently, we use Ganhand [27] to determine the optimal human hand grasping pose for the object. In the final stage, within the GraspIt! [28] collision detector, each feasible grasp is checked for collisions against the hand pose generated by Ganhand. We select the grasping pose with the highest score that avoids collisions with the human hand as the robot’s preferred grasping pose.

**Fig. 4.**
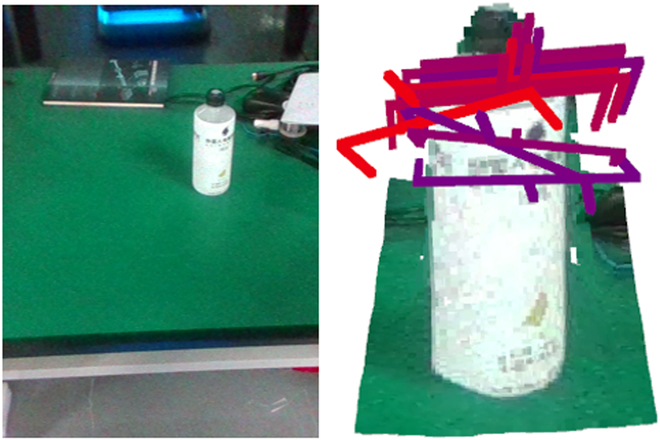
The figure depicts GraspNet’s grasp predictions for unfamiliar objects. The left displays onboard camera’s RGB data, while the right presents grasp predictions using RGB-D input.

Graspnet [16] is widely recognized for its robust grasping capabilities and has consistently showcased commendable performance across various grasping datasets. Notably, it can generate commendable grasping results, as depicted in Fig. 4, even for objects it has not been specifically trained on. By integrating depth maps with color images as inputs, GraspNet can end-to-end generate grasping poses for a two-fingered gripper. Owing to these strengths, we have adopted GraspNet as our grasp generator. This eliminates the need for specifying the object’s pose, thereby enhancing the adaptability of the entire system. Consequently, this flexibility means that objects can be placed arbitrarily without any predetermined orientation constraints.

We use a computer-aided design (CAD) model T shown in Eq. 1 and function W referenced in Eq. 2 to generate a math model N that provides a hand pose *P*, shape *V*, and grasp type *C* for an object in *T* and it called model parameterization:

N:T⟹C,V,P (1)

$$M\left(\theta ,\beta \right)=W\left(T\left(\beta ,\theta \right),J\left(\beta \right),\theta ,\omega \right)$$ (2)

**Fig. 5.**
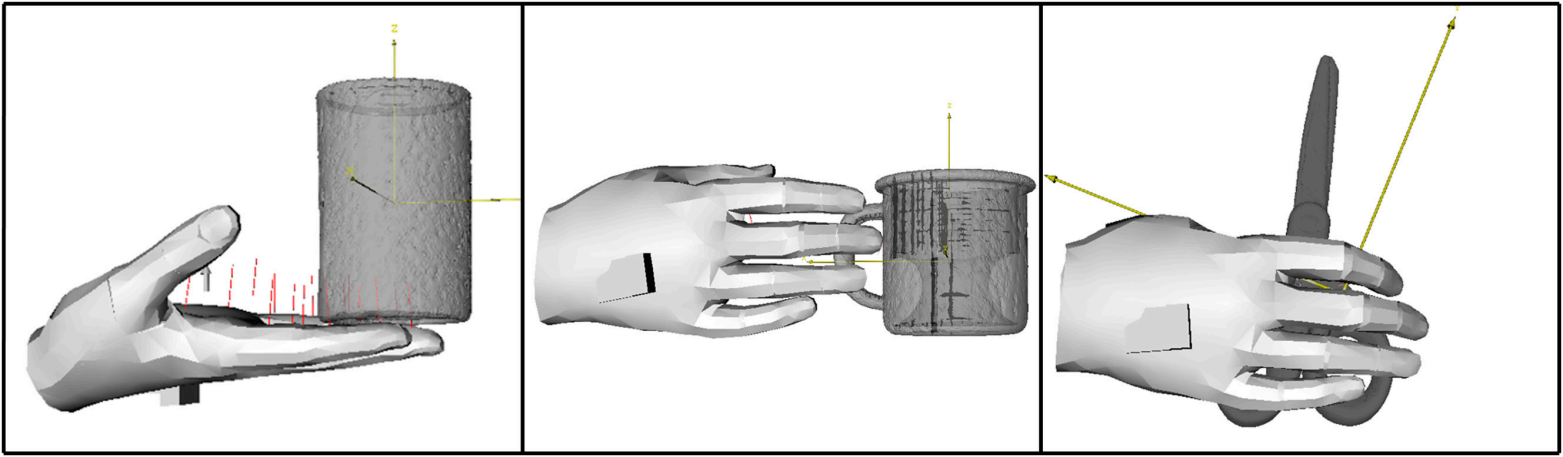
Appropriate grasp types of hand mesh generated on an object.

We have chosen Ganhand [27] as our hand generation method for three main reasons. First, Ganhand predicts the optimal human hand grasp type and generates a hand mesh on the object. Second, both Ganhand and our work utilize the YCB Dataset [29], allowing us to easily minimize the generalization error. Third, the authors use real objects grasped by human hands to train their model. For three objects from the YCB dataset, as shown in Fig. 5, optimal human hand grasp poses have been generated.

**Fig. 6.**
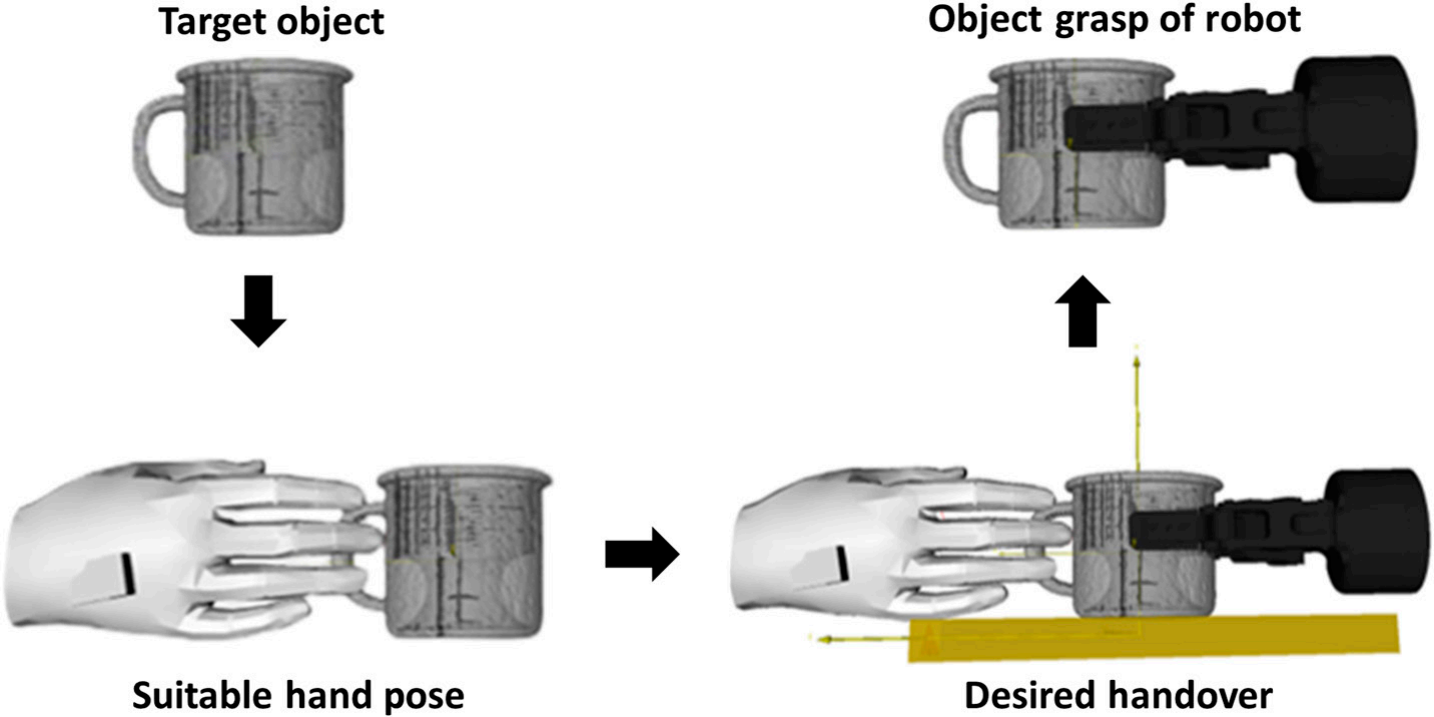
Given a target object, robot reconstruct the joints of the hand manipulating object. Based on the constrained object, Graspnet and GraspIt! create a safe and desired gripper pose. Finally, the robot solves the global pose through coordinate transformation and executes the planned trajectory.

Subsequent to generating the optimal human hand grasp poses, the object models overlaid with these poses are imported into GraspIt! [28]. It is a simulator equipped with a swift collision detection mechanism, tailored for formulating grasp poses based on the gripper’s design. It evaluates and scores each grasp pose generated by Graspnet, and the one with the highest score is selected as the desired end-effector pose for the robot. Our grasping methodology ensures not only a comfortable grasp for the human hand at the conclusion of the handover but also a collision-free successful grasp by the robot. Figure 6 illustrates the entire process of generating the grasp pose for the “mug” model from the dataset.

### Hand tracking and mesh generation

The visual data we utilize originates from a color and depth (RGB-D) camera. Each image pair is represented as $f_{i}=\left(C_{i},D_{i}\right)$. In this representation, $C_{i}:\Omega \to {\mathbb{R}}^{3}$ corresponds to the *i*th color image, while $D_{i}:\Omega \to \mathbb{R}$ represents the *i*th depth image. Here, Ω ⊂ *ℝ*^2^ stands for the image domain. Notably, the color image $C_{i}$ is employed to predict the hand skeleton.

For each human hand, a learning-based algorithm is employed for detection. This detection is subsequently refined by leveraging geometric relationships. We utilize MediaPipe [19], a renowned solution for precise hand and finger tracking. It capitalizes on machine learning to deduce information about hand joint points. Through this approach, we can real-time obtain 2D hand joint point pixels as (*u*_1_, *v*_1_), (*u*_2_, *v*_2_), …, (*u*_21_, *v*_21_). Notably, the numerical sequence for the joints aligns with the conventions laid out in [30].

**Fig. 7.**
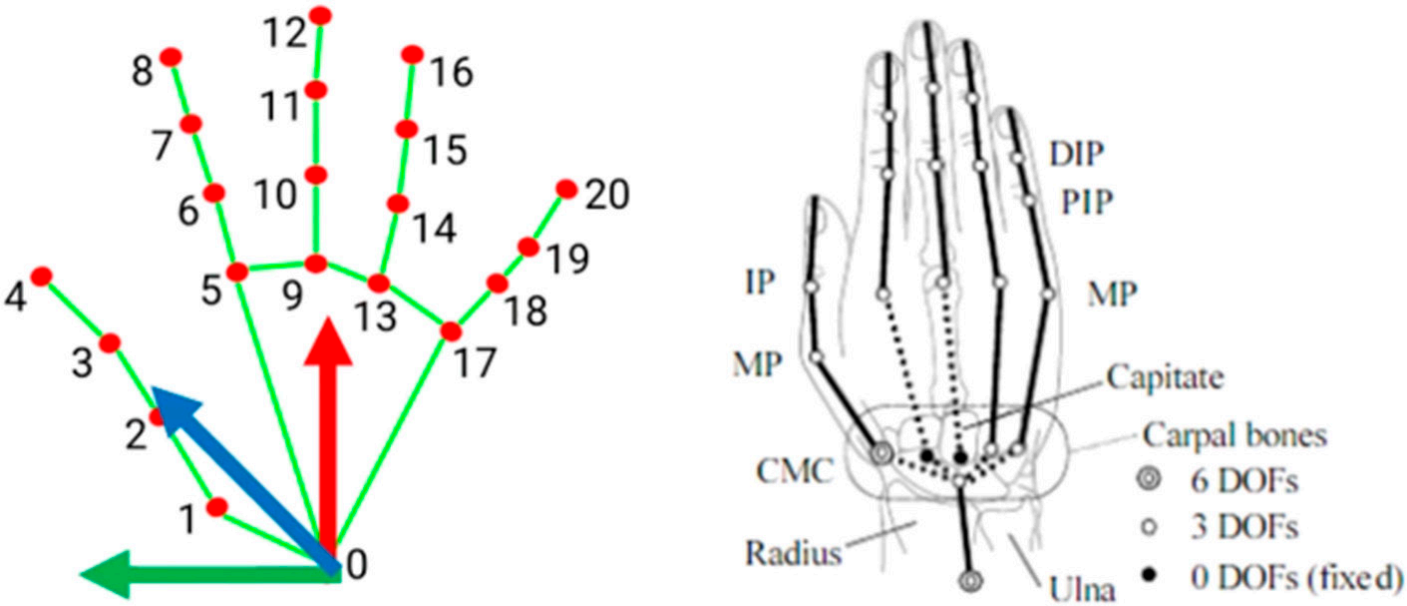
Diagram of human hand keypoints: The left side of the figure illustrates the numbering of the hand keypoints, with the orientation of the hand specified to be the inner side of the palm. The right side showcases the degrees of freedom for each joint of the hand.

The next step is to construct the coordinate transformation of the end effector coordinate system $F_{f}$, the camera coordinate system $F_{c}$, and the robot coordinate system $F_{r}$. Specifically, we obtain $F_{c}$ based on the intrinsic parameters of the RGB-D camera, $F_{f}$ and $F_{r}$ from unified robot description format (URDF) parameters and laser odom. However, since the robot requires a 6D target as motion planning inputs, we need to define $F_{h}$ to describe an arbitrary handover point for robot put the object. According to the article [31], the index finger root, ring finger root, and wrist have little deformation when human hand arbitrarily moves around deformably. Therefore, we select points *q*^0^, *q*^5^, and *q*^9^ to build the hand plane $S_{a}=\left[\;\vec{x},\;\;\vec{y},\;\vec{z}\;\right]$ and specify the inner side of the palm as the positive direction of the *z* axis shown in Fig. 7.

We examine the MediaPipe outputs, which consist of joint points *p_i_* that have a confidence *c* > 0.7 and are not self-occluded, represented as a set *C* = {*p*_1_, *p*_2_, …, *p_i_*}. The detection of self-occlusion serves to prevent erroneous depth correspondences for joint points. When two joint points share the same pixel depth, we retain the one closer to the fingertip and discard the one closer to the palm. We use the pin Eq. 3 to compute the 3D point relative to $F_{c}$. Here, *f_x_*, *f_y_*, *c_x_*, and *c_y_* are the intrinsic parameters of the camera, and *u_i_*, and *v_i_* are the pixel values corresponding to the *i*th point. Leveraging Eq. 4 and taking into account the hand keypoints represented in both the MediaPipe’s native frame and the camera’s frame, we derive the transformation matrix between these coordinate systems. This transformation facilitates the projection of $S_{a}$ onto $F_{r}$, thereby pinpointing the precise handover point for robot–object interaction.

$$p_{i}={\left(\frac{u_{i}-c_{x}}{f_{x}}d_{i},\frac{v_{i}-c_{y}}{f_{y}}d_{i},d_{i}\right)}^{T}$$ (3)

$$H=\sum_{i=1}^{n}\left(p_{i}-\frac{1}{n}\sum_{j=1}^{n}p_{j}\right){\left(q_{i}-\frac{1}{n}\sum_{j=1}^{n}q_{j}\right)}^{T}$$ (4)

We have opted for the MANO model [22,23] as the driving hand model and body model in our study. This choice is based on its demonstrated superiority in model-based hand and body reconstruction compared to model-free alternatives. The MANO model’s surface mesh can be fully deformed and posed using the standard linear blend skinning function. In Eq. 2, $M\left(\theta ,\beta \right)\in {\mathbb{R}}^{773\times 3}$ represents the hand mesh surface, while *β* ∈ ℝ^10^ and *θ* ∈ ℝ^16×3^ denote shape and pose parameters, respectively. Additionally, *ω* is a constant term. Our primary goal is to utilize *H_j_* to estimate both *β* and *θ* in a process known as hand inverse kinematics.

Manolayer [32] is a differentiable Pytorch layer that offers direct mapping from *β* and *θ* to M and joint positions. This design enables the leveraging of an automatic differentiation engine to optimize the parameters *β* and *θ*. We initiate this process by setting both parameters to zero and subsequently deriving the 3D coordinates of the hand joints via Manolayer’s forward channel. Treating $F_{\mathrm{rh}}$ and *H_j_* as ground truth, we compute the loss between these values and the output generated by Manolayer. Once the first frame is successfully initialized, we use the obtained *β* and *θ* as initial values for further optimization. Ultimately, we iteratively update *β* and *θ* in a reverse manner to generate the current hand mesh. The processes of generating human body and hand models are analogous. The primary objective of both models is to act as obstacles in the subsequent motion planning phase. This ensures that the robotic arm, when planning its path, opts for trajectories that avoid both the human hand and body, ensuring a smooth handover.

## Results

### Experimental platform and design

**Fig. 8.**
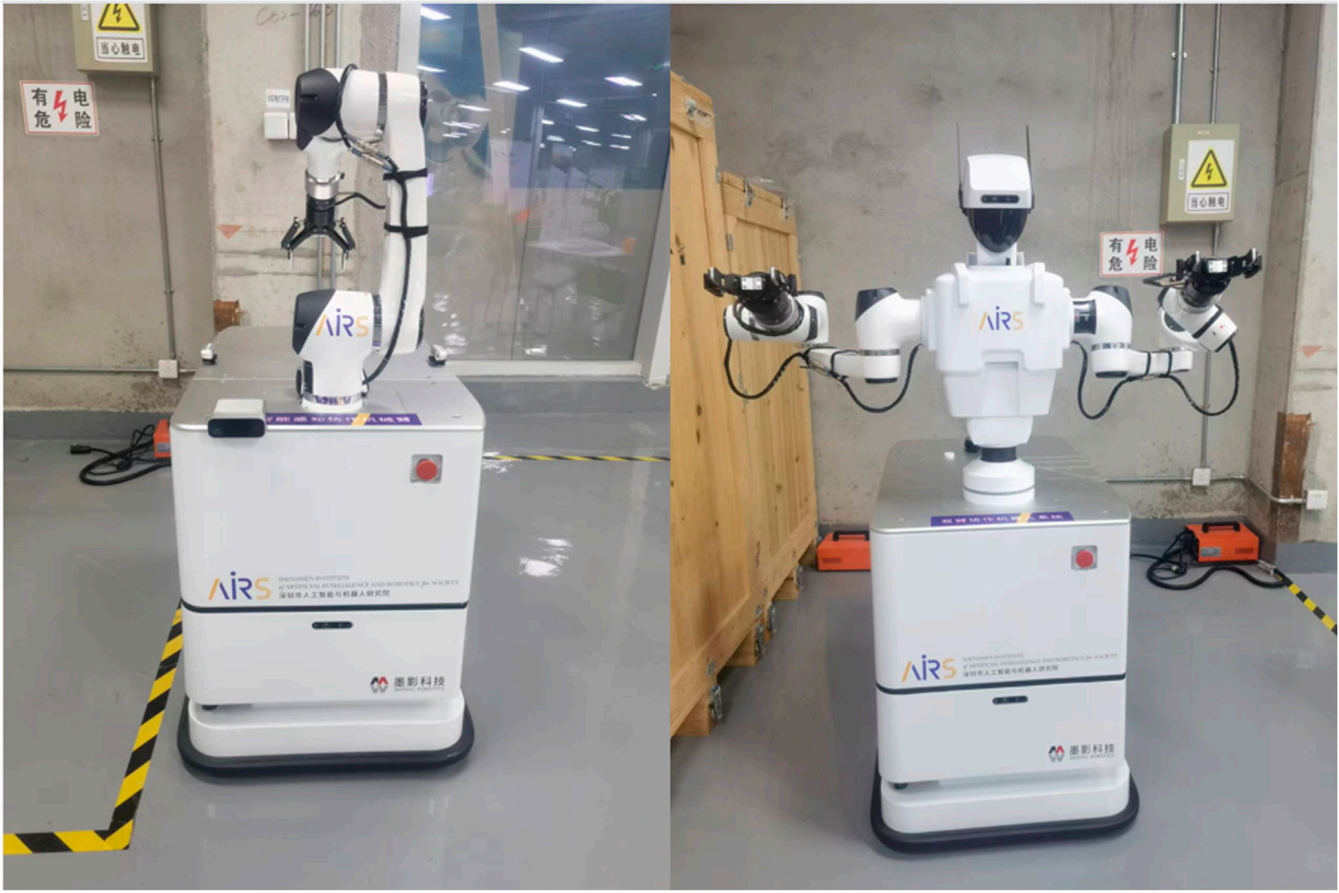
The single-arm robot with an Elfin5 arm and the dual-arm robot with an Elfin3 arm, equipped with advanced vision sensor and computational setups for handover tasks.

Within this section, we present the experimental platform and methodology, with accompanying video demonstrations provided in the Supplementary Materials. Our system was deployed and evaluated on two robotic platforms sharing a common chassis, each outfitted with a high-performance SICK 2D laser scanner. As depicted in Fig. 8, the single-arm robot is equipped with an Elfin5 robotic arm, and the dual-arm robot sports an Elfin3 arm, offering flexible and accurate manipulation capabilities, respectively. The single-arm robot boasts an extensive visual data capture setup, comprising a dual Intel RealSense D435 RGB-D camera configuration alongside two Azure Kinect RGB-D cameras. In comparison, the dual-arm robot employs a single Intel RealSense D435 RGB-D camera, tailored for efficient and precise object recognition and localization tasks. All sensors mounted on the robots have undergone a rigorous calibration process to assure the reliability of the data acquired.

Computational duties for motion planning and execution are shouldered by an i5-7500 processor integrated within the robots. The vision perception module is run on a high-performance laptop that harnesses the power of NVIDIA Jetson AGX Orin 64GB Development Kit, enabling real-time processing of complex visual inputs. Communication between computers is orchestrated via the widely adopted ROS framework, with wireless connectivity ensuring uninterrupted data exchange across a unified local area network.

To conduct ablation analysis, our integrated system is dissected into two main modules: Object Goal Navigation and Robot-to-human Handover. These components are subsequently benchmarked against prevailing methods in the field to assess their individual impact on system performance.

### Object goal navigation experiments

To assess the efficacy of our proposed navigation method, we have opted to compare it with traditional approaches rather than the current trend in object navigation. The rationale behind this decision stems from the specific requirements of our task: locating and retrieving a particular object placed on a table, as opposed to any arbitrary object within a room. Such specificity negates the need for a commonsense understanding of object semantics. This approach is deliberate as it circumvents the necessity for scene comprehension and segmentation, thereby conserving considerable computational resources.

In typical object navigation tasks, the system cannot operate in real time directly on the robot’s onboard processors due to the current limitations of cost-effective embedded artificial intelligence (AI) platforms commonly used in robots. These platforms usually struggle to concurrently run intensive processes such as SLAM (simultaneous localization and mapping), indoor semantic segmentation, and point cloud processing in real time. Testing is often conducted on pre-processed datasets focusing on individual modules rather than the entire integrated system.

**Fig. 9.**
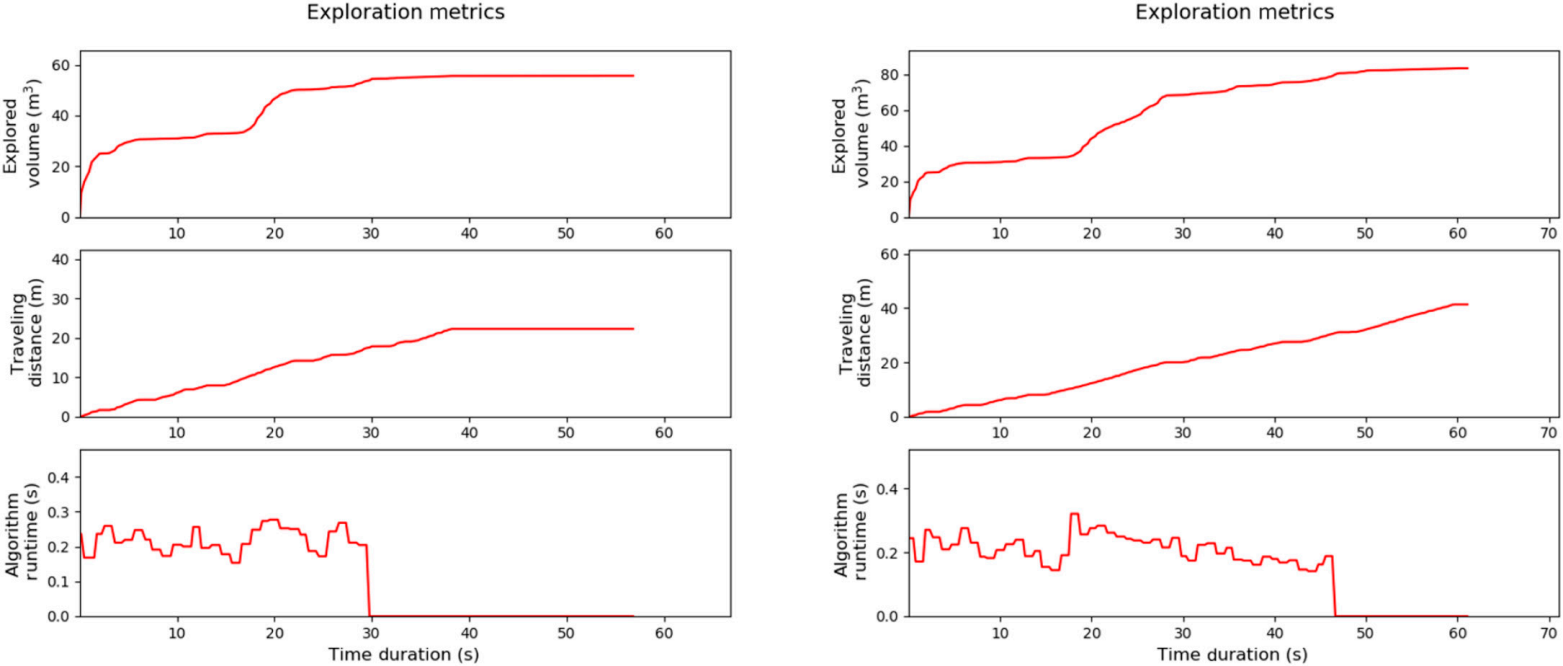
Performance comparison in Matterport3D environment: The figure illustrates a side-by-side comparison of volume coverage and completion time, where the left graph represents the baseline method, and the right graph corresponds to our proposed method [25]. It is evident that our method achieves superior volume coverage while maintaining competitive completion times.

Our objective is to cover the entire room and locate the object in the shortest possible time. To ensure a fair validation of our algorithm’s effectiveness, we conducted tests within the same simulated environment, measuring the time required to achieve complete coverage. This method provides a controlled benchmark for comparing our navigation strategy against established methods in a consistent and quantifiable manner. As illustrated in Fig. 9, the results were obtained from experiments conducted within the Matterport3D simulation environment. On the left, the baseline model is presented as described by Zhu et al. [25], upon which we have improved by modifying the RRT node generation strategy. Our enhancement strategically scatters observation points near large obstacles, thereby increasing the likelihood of observing otherwise concealed smaller rooms and enhancing both the volume and efficiency of area coverage. Given our objective to detect objects during the coverage process, the expansion of the covered area directly correlates with a higher probability of object discovery.

### Handover experiments

**Fig. 10.**
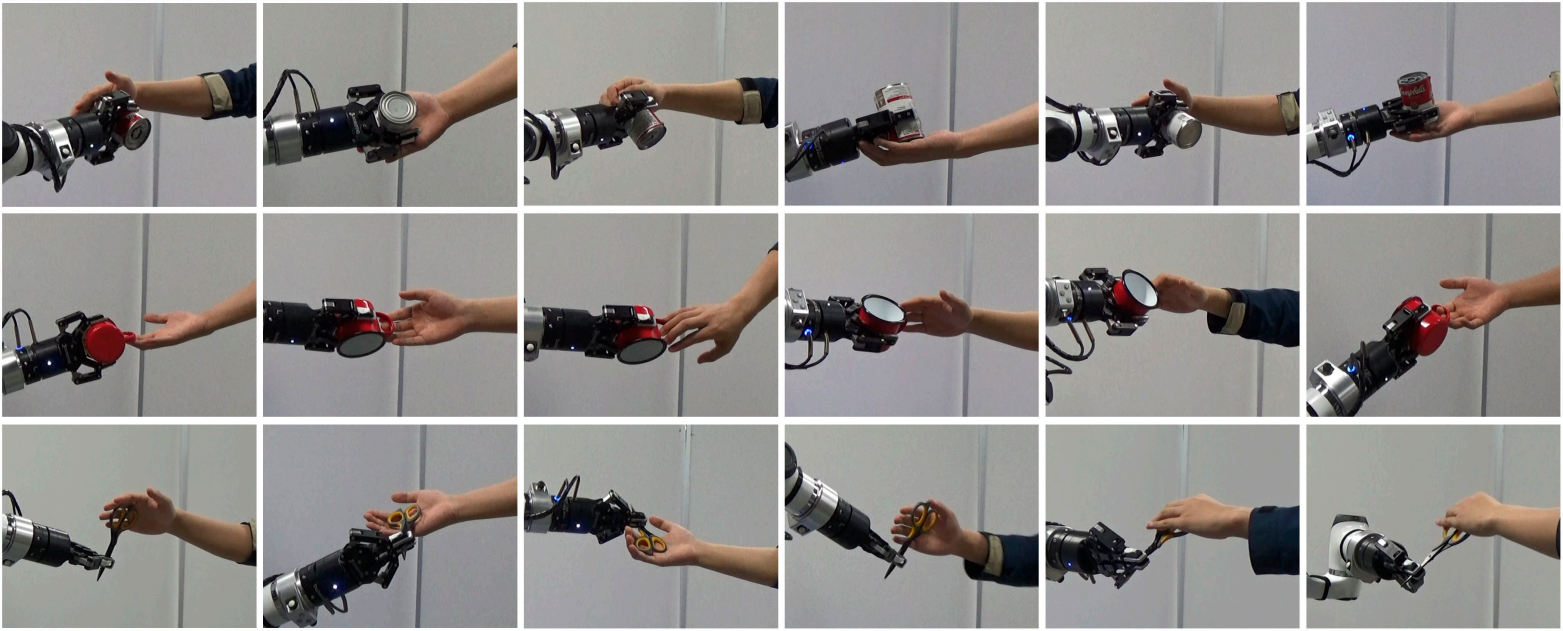
Handover success example. Three objects of different sizes and shapes were used: a can, a mug, and scissors. The same user performed all experiments. For any hand pose, the robot successfully delivered the object to the human.

We conducted experiments to test the performance of our system in response to different types of handover with arbitrary hand poses. Specifically, we looked at three different objects. As shown in Fig. 10, the objects were a mug, scissors, and a can from the YCB object-set [29] that fit the Moying robot gripper. For each object, we ensure that the human hand is within the workspace of the robot. The pose of the human hand can be arbitrary, and the robot needs to send the object to the human hand. When the position of the human hand changes, the robot needs to respond in time, adjust the posture of the end effector, and deliver the object to the human again. We conducted 30 experiments on each object. Each experiment is divided into three stages. The first stage is that the end effector sends the object to the human hand from the initial position. The second stage is that after the first movement of the hand, the manipulator will redeliver the object to the hand from the current position. The third stage is the same as the second stage. The purpose of this process is to test whether our system can cope with the problem of hand movement and arbitrary hand pose.

To ensure the fluency of people’s subjective experience, we need to set the maximum time for one delivery; if it exceeds this time, we judge the experiment to fail. We refer to the average time taken for a delivery in previous work to set the metric. Our metric is that a handover must be completed within 8 s and free collisions with the human hand. Some of our successful examples in the experiment are shown in Fig. 10. It can be seen that we realize handover based on arbitrary hand pose. For three object handover, the robot execution success rate and execution time are shown in Table 1. As can be seen from the data distribution in the table, the success rate of our system does not depend on the handover object.

**Table 1.** Success rate of 30 experiments per object in handover tasks

Object	Mug	Can	Scissors
Hand still	100%	96%	100%
First hand move	90%	86%	96%
Second hand move	76%	76%	86%

In order to substantiate the enhanced comfort and user-friendliness of our system, we have undertaken a comparative analysis with existing methodologies within the domain. In light of the paucity of studies on comfort assessment in robot-to-human handovers, our research conducts a comparative analysis with the seminal work of Aleotti et al. [33]. We have built upon the evaluation criteria established in their research to benchmark our system. Following a similar experimental setup, we recruited 10 participants at the Shenzhen Institute of Artificial Intelligence and Robotics for Society, comprising 6 males and 4 females, with ages ranging from 22 to 35 years (mean age of 25).

During the handover phase, there are several distinctions between our method and that of [33]. First, the handover point in their study is fixed, necessitating participants to move their hand to a predetermined position to receive the object. In contrast, our approach allows for arbitrary hand postures, requiring only that the hand is extended within the robot’s field of vision. Second, their system releases the object after visually detecting an overlap between the human hand and the object for 2 s, whereas our system relies on the force measured by torque sensors in the robot’s wrist to determine the handover.

Each participant conducted three trials per item: a human experimenter handing over an item to the participant, our system handing over an item, and the system from [33] doing the same. Participants were unaware of which system they were interacting with during each trial. Upon completion of the experiment, all participants were asked to fill out a questionnaire designed to assess the comfort level of the two systems. Table 2 presents the results on a scale ranging from 1 (strongly disagree) to 5 (strongly agree). We set the baseline for comfort as the experience of receiving an item from the experimenter, rated as 5, and asked participants to score the two systems in comparison. The data articulated in the tables of this section clearly indicate that our proposed method substantially enhances performance of comfort when juxtaposed with the comparative method while also closely emulating the optimal comfort experience of human-to-human handovers.

**Table 2.** Participants’ experience rating score

Question	Our method	Comparative method [33]	Human
Delivery time satisfaction	3.4 (±0.31)	1.2 (±0.62)	5
Safety satisfaction	4.0 (±0.43)	3.2 (±0.34)	5
Hand comfort satisfaction	4.4 (±0.55)	3.7 (±0.67)	5

## Discussion

We introduce a comprehensive handover framework tailored for mobile robots, designed to seamlessly manage the entire handover process, from object location to grasping and final delivery. A prominent feature of our framework is its advanced vision-based system that is adept at recognizing and interpreting a wide range of human hand postures through a specialized detection and reconstruction approach. This allows for accurate estimation of hand poses in various configurations. The framework’s ability to identify the optimal grasp type is crucial, as it ensures that the robot can determine both a safe hand posture for the human recipient and a successful grasp configuration for the object itself. To demonstrate the versatility and effectiveness of our system across different scenarios, we have rigorously tested our robot-to-human handover algorithm on both single-arm and dual-arm robots. The results, showcasing the algorithm’s adaptability and performance in varied settings, are presented in a video included in the Supplementary Materials. This evidence further substantiates the robustness and general applicability of the algorithms constituting our handover system.

**Fig. 11.**
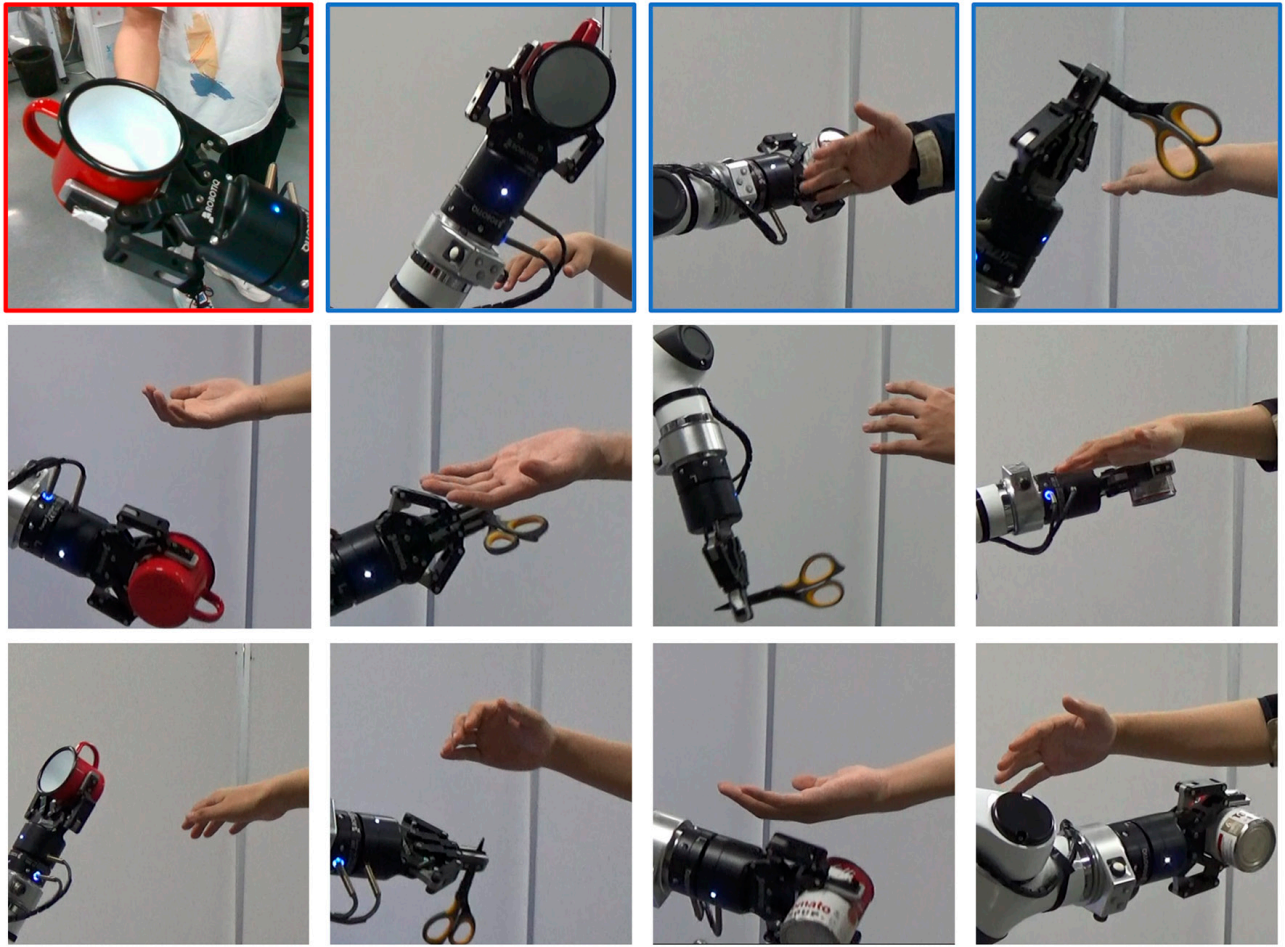
Handover fail examples in our experiment. The failure case with a red border means that the camera is blocked by an object and can not find a human hand in the new position. The blue border means that the human hand and robotic arm move at the same time and occlude the camera. Other means the path is too long to deliver the object within the allotted time.

As shown in Fig. 11, the failure case with a red border means that the camera is blocked by an object and can not find a human hand in the new position. The blue border means that the human hand and robotic arm move at the same time and occlude the camera. Other means the path is too long to deliver the object within the allotted time. There are two reasons for the 20.7% failure rate after two hand position changes. First, the planning algorithm we use is not globally optimal, because the globally optimal planner will consume too much time, which will affect people’s subjective feelings. Exceeding the specified time in our experiments will be considered a task failure. At the same time, due to the prevention of collision with the human hand, we cannot just use the inverse kinematics algorithm to solve the desired pose. So we choose RRT-connect to reduce planning time. However, this algorithm does not guarantee the globally optimal path, which means that the path will become longer, which will also lead to timeouts.

Second, after the human hand moves, some parts of the robot arm may occlude the human hand in the new position, causing the robot to fail to correctly estimate the pose of the human hand. Besides, when the robotic arm delivers the object, it just blocks the moving hand and cannot stop according to the reactive strategy. However, our system does not cause harm to people in these failure cases, such as collision with a human body or hand. Our next work will also focus on solving the problems in these three aspects to achieve a safer, faster, and more comfortable robot-to-human handover.

Besides, we must consider the constraints imposed by the robot’s sensors and computational capabilities. Limitations of vision-based techniques primarily manifest in the depth accuracy of RGB-D cameras, particularly in challenging conditions such as bright lighting or outdoor environments, where depth perception can significantly impact the precision required for handover tasks. The accuracy of human hand skeleton prediction is largely dependent on the distance from the camera to the subject; beyond a certain range, it becomes challenging to predict the skeletal structure of the hand effectively. Therefore, a local planner is necessary to move the robot closer to the person and position it to face them. While adjusting the angle of the robot’s head joint can expand the field of view, our study does not account for the control of head joints, potentially leading to situations where the human hand may not be within the camera’s viewing range.

Our computational resources are based on the NVIDIA Jetson Orin platform. The consumption of computational resources is primarily in three areas:

• Elevation map processing: This requires the use of a GPU for parallel point cloud computation, which can achieve higher frame rates compared to CPU-based elevation map processing.

• Detection of humans and objects: This task necessitates the processing of high-frame-rate images with a GPU to meet real-time requirements.

• Reconstruction of human hand mesh: The use of PyTorch’s automatic differentiation is necessary, and for the real-time requirements of downstream motion planning, GPU execution is also required.

The data that support the findings of this study are available from the corresponding author upon reasonable request. During testing, these computational demands could be simultaneously met. In contrast, the work [20] involved tasks such as human-to-robot handover, which employed neural networks for human body segmentation and hand segmentation. Their system implementation, distributed across five computers with Nvidia GPUs with at least 8 GB of memory, could not fulfill the requirements for Embodied AI tasks. This limitation also contributes to our decision not to use scene segmentation and reinforcement learning-based decision-making within the Object Goal Navigation component.

## Data Availability

All data needed to evaluate the conclusions of this study are available within the paper. There are no restrictions on data availability.
